# Understanding the role of intersectoral convergence in the delivery of essential maternal and child nutrition interventions in Odisha, India: a qualitative study

**DOI:** 10.1186/s12889-017-4088-z

**Published:** 2017-02-02

**Authors:** Sunny S. Kim, Rasmi Avula, Rajani Ved, Neha Kohli, Kavita Singh, Mara van den Bold, Suneetha Kadiyala, Purnima Menon

**Affiliations:** 1 International Food Policy Research Institute, 2033 K Street, Washington, D.C., 20006 NW USA; 2 International Food Policy Research Institute, NASC Complex, CG Block, Dev Prakash Shastri Road, Pusa, New Delhi, 110012 India; 3 National Health Systems Resource Center, National Institute of Health and Family Welfare campus, Baba Gangnath Marg, Munrika, New Delhi India; 4 London School of Hygiene & Tropical Medicine, 133b LSHTM Keppel Street, WC1E 7HT London, England

**Keywords:** Collaboration, Coordination, Intersectoral convergence, Nutrition interventions, India

## Abstract

**Background:**

Convergence of sectoral programs is important for scaling up essential maternal and child health and nutrition interventions. In India, these interventions are implemented by two government programs – Integrated Child Development Services (ICDS) and National Rural Health Mission (NRHM). These programs are designed to work together, but there is limited understanding of the nature and extent of coordination in place and needed at the various administrative levels. Our study examined how intersectoral convergence in nutrition programming is operationalized between ICDS and NRHM from the state to village levels in Odisha, and the factors influencing convergence in policy implementation and service delivery.

**Methods:**

Semi-structured interviews were conducted with state-level stakeholders (*n* = 12), district (*n* = 19) and block officials (*n* = 66), and frontline workers (FLWs, *n* = 48). Systematic coding and content analysis of transcripts were undertaken to elucidate themes and patterns related to the degree and mechanisms of convergence, types of actions/services, and facilitators and barriers.

**Results:**

Close collaboration at state level was observed in developing guidelines, planning, and reviewing programs, facilitated by a shared motivation and recognized leadership for coordination. However, the health department was perceived to drive the agenda, and different priorities and little data sharing presented challenges. At the district level, there were joint planning and review meetings, trainings, and data sharing, but poor participation in the intersectoral meetings and limited supervision. While the block level is the hub for planning and supervision, cooperation is limited by the lack of guidelines for coordination, heavy workload, inadequate resources, and poor communication. Strong collaboration among FLWs was facilitated by close interpersonal communication and mutual understanding of roles and responsibilities.

**Conclusions:**

Congruent or shared priorities and regularity of actions between sectors across all levels will likely improve the quality of coordination, and clear roles and leadership and accountability are imperative. As convergence is a means to achieving effective coverage and delivery of services for improved maternal and child health and nutrition, focus should be on delivering all the essential services to the mother-child dyads through mechanisms that facilitate a continuum of care approach, rather than sectorally-driven, service-specific delivery processes.

**Electronic supplementary material:**

The online version of this article (doi:10.1186/s12889-017-4088-z) contains supplementary material, which is available to authorized users.

## Background

Despite India’s impressive economic growth, undernutrition is widespread in the country amongst all ages. Nearly 30% of Indian children under 5 years are underweight, 39% are stunted, 15% are wasted [1], and over 70% are anemic [2]. More than half of all women of reproductive age are anemic [2]; yet, only 31% of women received any iron and folic acid supplements [1]. Less than one-third of the children aged 6–23 months received adequate complementary feeding, i.e., the minimum number of food groups (20%) or the minimum meal frequency (36%) [1]. Interstate variability of health and nutrition indicators also exist [3], with disparities in health systems between and within states [4], calling for the need to address delivery gaps and increase coverage of essential interventions to reach all target populations.

Increasing the coverage of the nutrition interventions already in place in countries has been suggested to markedly reduce maternal and child undernutrition [5]. These include interventions such as nutritional counseling and food and micronutrient supplementation for women and young children at different life stages - before and during pregnancy, newborn to infancy period, and early childhood. Despite a strong consensus on what needs to be done, less is known about how to operationalize the mix of actions required for scaling up, wherein convergence of sectoral programs plays an important role [6].

Recent studies have described processes of multisectoral coordination in various countries, identifying challenges and key factors for successful coordination. A five-country study showed that differences in institutional mandates lead to lack of sound coordination mechanisms, and dissent among mid-level actors in formulating and agreeing upon different intervention strategies are common barriers; leadership, defined roles and responsibilities, and individual and strategic capacity are important to overcome such challenges [7]. A qualitative institutional study of national policy-making in four Sub-Saharan African countries observed that policies and agencies that have cross-sectoral scope do not usually fit the sectoral pattern of resource allocation, thus the ministries may view themselves as in competition with each other [8]. High-level political support and processes that bring together a wide variety of stakeholders [9] as well as shared vision, capacity strengthening, joint accountability, and supervision [10] are critical for multisectoral convergence. While multisectoral coordination and policies are generally viewed as valuable and important, subsequent actions and implementation of such policies require clearly defined methods and mechanisms at each administrative and operational level, particularly for integrative processes to be carried out in service delivery. There is, however, scarce literature on how convergence is operationalized and the mechanisms required to ensure effective service delivery.

In India, two ministries - the Ministry of Health and Family Welfare (MoHFW) and the Ministry of Women and Child Development (MoWCD), share responsibility for the implementation of all the essential nutrition interventions [11, 12]. Two programs, the National Rural Health Mission (NRHM), or the National Health Mission under the MoHFW, and the Integrated Child Development Services (ICDS) under MoWCD, aim to improve maternal and child nutrition and health, through services provided by their respective cadres of frontline workers (FLWs), who are expected to work together to deliver the interventions. Integrated governance structures between the health and nutrition departments were documented to be important for improved community participation, accountability of the public system, and service delivery [13]. However, operational inconsistencies exist between ICDS and NRHM, specifically in the management of severe malnutrition [14]. In the state of Odisha, political will, committed policy makers, and fiscal space spurred the health system to innovate and expand health service provision, reform health resource management and development, and introduce initiatives to achieve greater equity among the poorest and disadvantaged population groups [15]. Our previous document review [11] also revealed that national and state (Madhya Pradesh and Odisha) policy and program documents from the health and WCD sectors demonstrate common recognition of the importance of nutrition and consensus regarding the inputs necessary to address child undernutrition, but there are gaps in providing operational guidelines [16].

In this paper, we examine how intersectoral convergence in policymaking and programming is operationalized between the health and ICDS programs from the state to village levels in Odisha, one of the poorest states in India, where steps have been taken to enhance coordination between the NRHM and ICDS, particularly through inter-departmental coordination meetings and focus on intra-ministerial capacity strengthening [12, 17]. We aim to provide insights into how and why intersectoral convergence does or does not take place at different levels of policy implementation and service delivery, and the elements that are needed to effectively do so.

## Conceptual framework and definitions of convergence

**Fig. 1:**
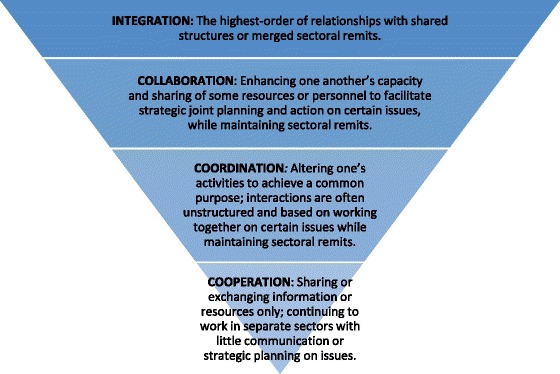
Degrees of convergence and their definitions

Frameworks on integration from organizational theory and other fields concur that degrees of integration exist as a continuum or process of evolution that modify over time [18–20]. Terms such as integration, collaboration, coordination, and cooperation are used interchangeably or defined differently to describe stages along the continuum [9, 18]. In this paper, we use the term “convergence” generally, as being synonymous with the overall continuum of integration. According to Axelsson and Axelsson [21], coordination, cooperation, and collaboration are usually placed somewhere in the middle of this continuum, between consultation and consolidation [of organizations]. Adapted from previous works on multisectoral approaches in nutrition by Garrett and Natalicchio and others [9, 22], we use the following working definitions for integration, collaboration, coordination, and cooperation in this paper (Fig. 1).

*Integration*: the highest-order of relationships with shared structures or merged sectoral remits. *Collaboration*: enhancing one another’s capacity and sharing of some resources or personnel to facilitate strategic joint planning and action on certain issues, while maintaining sectoral remits. *Coordination:* altering one’s activities to achieve a common purpose; interactions are often unstructured or based on a loose goal-oriented agreement and working together on certain issues while maintaining sectoral remits. *Cooperation*: sharing or exchanging information or resources only; continuing to work in separate sectors with little communication or strategic planning on issues.

## Methods

This study is part of a larger mixed-method operational research on intersectoral convergence and delivery of and exposure to essential nutrition interventions in Odisha state [23]. This paper draws primarily on results of the qualitative study conducted between July and August 2013 and data based on semi-structured interviews with stakeholders involved with the health and ICDS programs, as well as from other sectors (i.e., non-governmental organizations, multilateral agencies, and academia), at the state, district, block, and frontline levels.

### Sampling and sample size

Maximum variation sampling using a combination of purposive and random selection at different levels was applied to capture heterogeneity in service delivery contexts [24]. Three districts were purposively selected from among the 30 total districts in the state, to attain variations between districts. Existing district-level survey data [25, 26] were used to construct a set of criteria pertaining to service coverage and household factors (e.g., coverage of immunization and vitamin A supplementation, three or more antenatal care visits, and institutional delivery; access to toilet and electricity; and type of cooking fuel), and changes in these indicators between two survey rounds were examined. All districts were grouped into three categories: better performing districts (i.e., those with positive change over time), average performing districts (i.e., no change), and poorly performing districts (i.e., those with negative change). Then, in a meeting organized by the researchers to explain the study and conduct a joint process of district selection, state-level officials from the Department of Health and Family Welfare (DHFW) and the Department of Women and Child Development (DWCD) randomly drew the name of one district from each category: District 1 as better performing, District 2 as average performing, and District 3 as a poorly performing district.

In each district, we randomly selected four blocks (subdivision or town) (*n* = 12) and two villages from two of the blocks (*n* = 12) to study service delivery by three different cadres of FLWs within each village. In a village, there is usually one Anganwadi Center (AWC) with an Anganwadi Worker (AWW). The AWW is responsible for the AWC and delivers health and nutrition services and preschool education under the ICDS program; she is an honorary female worker selected from the local community. The AWW in each village, along with the Accredited Social Health Activist (ASHA) and the Auxiliary Nurse Midwife (ANM) working in the same area were interviewed in this study. The ASHA is a female health worker, selected from the community and serves the role of community mobilizer to access services and community-level care provider. The ANM is a multipurpose female health worker who provides a package of preventive and curative services largely for women and children, at a sub-center that covers a population of 3,000–5,000 for rural areas, which is approximately 6–8 villages, and through outreach services. We conducted a total of 145 semi-structured interviews with the three types of FLWs, as well as with block, district, and state-level staff of the ICDS and health departments and from other sectors (Table 1). Interview guides are shown in Additional files 1, 2, 3, and 4.

**Table 1:** Study sample size by administrative level and sector

Level	Health	ICDS	Other sectors
State	4	1	7 (NGO, multilateral agency, academia)
District	11	6	2 (District collector^a^, GKS/VHSC^b^)
Block	23	32	11 (Block development officer^c^)
Village/Frontline	24 (ANM, ASHA)	12 (AWW)	12 (PRI^d^)
Total:	62	51	32

^a^District collector is the chief administrative and revenue officer, appointed by the state government

^b^ *Gaon Kalyan Samiti*/Village Health and Sanitation Committee (GKS/VHSC) is the local management body instituted by the National Rural Health Mission, comprised of village representatives and headed by a village ward member. GKS is responsible for community-based planning and implementation of health and related activities, and creating awareness and promoting public health and sanitation activities. It receives an untied grant of Rs. 10,000 annually (approximately USD 160) to ensure that such activities are carried out

^c^Block development officer is responsible for monitoring the implementation of all programs related to block planning and development

^d^Panchayai Raj Institution (PRI) is the oldest system of local government, the most basic administrative unit or assembly of community representatives, that is responsible for all matters of community development

Written informed consent was obtained from the study participants prior to interview. This study was approved by the IFPRI Institutional Review Board and Odisha’s Department of Women and Child Development and Department of Health and Family Welfare in India.

### Data analysis

Interviews were conducted in the local language (Odiya) and audio-recorded, then transcribed verbatim and translated into English. Extensive field notes were taken only for state-level interviews. We used a deductive approach to data analysis, applying pattern, theme, and content analysis for data reduction and to elucidate any similarities and differences and patterns of coordination between the ICDS and health programs for the interventions of interest [24]. A systematic process of data organization and analysis was led by the first author. The first level of analysis involved data coding using NVivo, a qualitative data software for computer-assisted data management and analysis. An *a priori* code list was prepared based on the study protocol and interview guides and tested using an initial set of transcripts. Then, a team of four coders (two coders were involved from the training of interviewers and data quality monitoring to summarizing the data) were trained to standardize the coding process, inter-coder reliability was tested, and the code list was reviewed and revised by the coding team and senior researchers. Bimonthly/monthly Skype calls were held to review coded data, any coding issues, and updates to the code list. Outputs of coded results using queries (on types of services, coordination mechanisms, facilitators and barriers) were generated by transcripts pooled at the district, block, and village levels. Then, second-level coding and summarizing for emergent themes and patterns were conducted. Among the various nutrition interventions that involve both the DHFW and DWCD, we present results on five intervention or service types (including combined interventions that are delivered simultaneously) to illustrate convergence in implementation: antenatal care (ANC) services, including maternal iron and folic acid (IFA) supplementation, child immunization and vitamin A supplementation, pediatric IFA supplementation, infant and young child feeding (IYCF) counseling/education, and growth monitoring and referrals for severe acute malnutrition. There were several driving questions for data analysis: To what extent do actors work together (degree of convergence), and how (mechanisms of convergence); on what do they work together (types of actions or services); and why (facilitators and barriers)? Also, are there any similarities or differences among the districts, blocks, or villages?

## Results

In our study, we found that convergence is operationalized to a different degree at the various levels of the government health and ICDS systems, i.e. from state to district, block and frontline levels. This may be expected, given the varied types of functions and relationships required at the different administrative levels. There are also key mechanisms through which actors interact or work together and factors that influence interactions in these mechanisms, which differ by levels (Table 2). These elements are elaborated in context in the subsequent sections of results.

**Table 2:** Summary of roles, degree and key mechanisms of convergence, and salient factors by different administrative levels

Level	Main role/action	Convergence degree and key mechanism	Salient factor: (+) facilitators and (−) barriers
State	-Establish state-wide programs and initiatives -Provide guidelines -Monitor and assess data -Allocate resources	*Collaboration*: -Developing guidelines -Meetings to discuss topics and plan and review programs and initiatives	(+) Shared motivation/goals (+) Recognized leadership for coordination (−) Different priority actions (−) Little data sharing (−) Lack of accountability and feedback mechanisms
District	-Prioritize services and activities -Plan annually/monthly -Monitor data reports -Allocate resources -Train block staff and FLWs	*Coordination*: -Planning and review meetings -Data sharing -Joint training sessions	(+) Clear leadership (+) Mutual understanding of roles (−) Narrow priority topics related to health and disease (−) Low participation/poor attendance (−) Limited supervision
Block	-Plan annually/monthly -Gather data records and registers and report -Supervise and feedback -Train/orient FLWs	*Cooperation*: -Planning and supervision	(+) Shared motivation (−) Lack of direction or guidelines (−) Heavy workload (−) Inadequate resources (−) Poor communication
Village/Frontline	-Schedule and implement services and activities -Record/register and report -Build rapport and demand creation in community	*Collaboration*: -Delivery of services, through VHND and home visits	(+) Shared motivation (+) Close inter-personal communication and vicinity (+) Understanding of roles and responsibilities (−) Unbalanced incentives

### Collaboration in setting policies and program guidelines at the state level

Stunting, wasting, and anemia were identified as the main nutritional problems in Odisha by nearly all state-level respondents. These problems were considered to be further exacerbated by challenges in the delivery and use of essential health and nutrition services, such as low registration of pregnant women and young children, inadequate access to food due to gaps in the supplement distribution, poor exclusive breastfeeding and complementary feeding practices due to inadequate counseling, and lack of adequate sanitation and hygiene. The state has taken various actions involving intersectoral collaboration in response to these challenges, including adapting the 2009 national guidelines for Village Health and Nutrition Days (VHND) (a monthly outreach program held at the AWC for integrated health and nutrition services for pregnant and lactating women, adolescent girls, and children under five years of age, and managed by all three FLWs) to state needs and naming it *Mamata Divas*. In 2011, the state government initiated the MAMATA program (a conditional cash transfer scheme that provides pregnant and lactating women with monetary support to improve nutrition and health-seeking behaviors that requires coordination among all three FLWs). Furthermore, guideline development for community-based management of severe acute malnutrition, as well as training of officials and service providers on the importance of and interventions for the “first 1,000 days” has been a joint effort between Odisha’s DHFW and DWCD.

Respondents concurred that improving maternal and child health and nutrition across the state is a shared goal and the joint responsibility of DHFW and DWCD, in addition to other departments. Several mechanisms were in place to facilitate regular coordination and collaboration, such as monthly meetings convened by (and perceived as dominated by) Health, and biannual project meetings, held to plan for specific programs or activities and convened by Health but chaired by the Development Commissioner to facilitate horizontal collaboration. Other examples included the cross-sectoral coordination committee for NRHM’s urban health program and the frequent cross-sectoral collaboration on guidelines for specific initiatives. Within these different meetings and activities, well-positioned leadership (or champions for the initiatives or issues) was seen as a key facilitator of convergence, particularly leaders involved not just in the line departments but who transcended departmental boundaries such as the Development Commissioner and the Chief Secretary.

Despite these successes, however, challenges to convergent actions remain. For example, despite the shared goal to reduce infant and maternal mortality, DHFW focuses on antenatal care services and DWCD contributes to improving maternal nutrition by providing food supplements during pregnancy, and there is little data sharing across these actions to demonstrate process towards the goal, even where common indicators exist. This has resulted in discrepancies in data presentations and limitations on the extent to which there is collaboration on program monitoring. Although policies and platforms for integrated service delivery have been developed, there is little evaluation and resource allocation to understand the performance of these strategies. There is also limited supervision and lack of accountability mechanisms for the implementation process from the state to lower levels, which leads to repercussions (such as irregular meetings and poor attendance) as discussed in following results.

### Coordination in planning, training, and data sharing at the district level

At the district level, DHFW and DWCD staff clearly identified their roles and responsibilities as applying state guidelines for programs, prioritizing services or activities based on their contexts, planning, monitoring data, allocating resources, and training block-level staff. District level officials primarily coordinated across sectors through review meetings and training sessions for local staff on specific common priority actions. However, the extent and nature of coordination between DHFW and DWCD at the district level was dependent mainly on the specific intervention types and narrow outcomes of interest in common to the individual departments.

There was good coordination in implementing the immunization program in all three districts. The departments often came together to prepare annual or monthly action plans, as a district program officer (ICDS) explained: “*There is an immunization plan for the entire district*, *which is the joint action plan of health and ICDS departments. It is prepared with coordination between both the departments… so that service is provided in a better way*, *coverage is expanded*, *coordination is maintained*, *and there are no missed cases.”* District coordination meetings were also held for special programs such as pulse polio and vitamin A supplementation campaigns. Additionally, the departments coordinated for training about IYCF, use of maternal and child (health) protection cards, and MAMATA program guidelines. Coordination at the district level was reported to be driven by directives issued from the state.

In each district, monthly review meetings are convened by the chief administrative official, i.e., the state-appointed district collector. During these meetings, ICDS and health staff reported their activities, reviewed outcomes, and assessed any gaps. The topics discussed most often were infant and maternal mortality, immunization, VHND, diseases and other health topics usually decided by health staff. While most of the district-level respondents considered these meetings as necessary, poor participation or low attendance were identified as common problems: “*Attending the coordination meetings should be regular. If [staff] regularly participate in those meetings*, *we will know the gaps and take steps*… *but they are not regular*, *so we face some difficulties.”* (District community mobilizer, Health). However, little accountability and consequence were seen for participation in meetings. Data sharing also takes place at the district level, but a narrow set of indicators was identified across all three districts, particularly infant and maternal mortality, verbal autopsy, institutional delivery, and immunization.

Supervision was reported to be limited, and when it does take place, it focused mostly on immunization and VHNDs or addressing problems or emergency cases. Lack of time was named as the main barrier to conducting joint supervision. Only in District 2, joint supervisory visits and coordination of supervisory activities was frequently reported, as described by one district health official: “*When I go to an Anganwadi centre*, *I always check the registers of that Anganwadi*, *weights of the children*, *food distribution*… *I also monitor the instruments*, *logistics*, *infant registry*, *and birth registry. When they* [*ICDS officials] visit*, *they look into both ICDS and health activities*, *and whenever I visit*, *I also check both activities*.”

### Limited cooperation for supervision at the block level

The main responsibilities at the block level included planning activities, monitoring and reporting data, and supervision. “*Both departments* [*DHFW and DWCD] are very much associated with each other because they have the same objectives*.” (Block program manager, Health). However, most block-level respondents expressed limited cooperation overall. “*Each person is doing his or her responsibilities very well. There is no need of any help* [*from each other*]. *We are aware about* [*each other’s*] *programs*, *but we are doing our work*, *and they are doing theirs*” (Lady supervisor, ICDS). Block-level respondents pointed to the lack of direction or guidelines for intersectoral coordination from higher levels, thus it was unclear how they were expected to work together.

Staff worked across sectors primarily for meetings and supervision. Separate ICDS and health sector meetings are held regularly and attended by respective block-level and field staff. Although staff from the other department are usually invited to participate at the sector meetings, to learn about planned activities and any outcomes and gaps, cross-sectoral participation was low and irregular. The lack of time or heavy workload, schedule conflicts, lack of physical space to conduct joint meetings, and poor communication were named as barriers. “*They* [*health staff*] *do not attend ours* [*sector meetings*], *and we do not attend their meetings. Because it is a fixed date*, *when they do not have a meeting and have time*, *they attend our meeting. And when we don*’*t have any*, *we attend their meeting*,” explained a child development project officer (ICDS). One public health extension officer shared, “*I have the letter from ICDS*, *that they asked me to come on a particular date*, *and I made plans and sent it to the CDPO* [*child development project officer*]…*but that day there was no such meeting*, *so my staff returned and complained that you sent us for a meeting but there was no meeting*… *they make such changes without any notice. We do not know the date of their plans*.” Similar to the district level, the topics discussed were limited to immunization and infant and maternal mortality, as data for these topics (e.g., tally of birth and death cases) are collected by both sectors.

Extent of coordination or joint supervision visits and frequency of supervision varied only slightly among blocks in different districts. Despite expressing the lack of guidelines for coordination in supervision, block staff usually planned together to cover supervision for immunization and VHND. Joint supervision involving staff from both departments was more frequently reported in Districts 1 and 2, particularly for verification of maternal or infant deaths and in case of emergencies or field problems. However, lack of time or heavy workload and insufficient resources (i.e., personnel and vehicle for transport, particularly in District 3) were repeated as barriers to coordination. “*There is good relationship*, *but due to paper work and other work*, *we cannot attend their meetings or supervision*.” (CDPO). In various blocks, there were reports of vacant posts or insufficient staff allocation and vehicles for conducting supervision. A public health extension officer explained, “*I supervise 25 ANMs in 25 sub-centers*… *in the block*, *there are 4 supervisors for 8 sectors*, *not enough*, *and 100 plus AWWs and 60 ASHAs in the villages*.” Among the administrative levels, most gaps and limitations in convergence appeared at the block level.

### Close collaboration for service delivery in the frontlines

FLW functions involved planning and implementing services and activities, maintaining data records, submitting reports, and building rapport with communities, particularly to create demand for services. All of the FLWs across the districts described working together, facilitated by their close vicinity (AWWs and ASHAs usually live within the same village) and interpersonal relationship.

With overlapping functions and activities, the three cadres of FLWs work together to deliver nearly all of the essential nutrition interventions. We present findings on FLW roles and factors influencing the provision of five specific interventions to illustrate the extent of convergence among FLWs (Table 3).

**Table 3:** Examples of roles and factors influencing collaboration among frontline workers, by intervention type and district

Interventions (Exposure^a^, %)	District 1		District 2		District 3	
	Understanding of roles and responsibilities	Factors enabling or hindering collaboration	Understanding of roles and responsibilities	Factors enabling or hindering collaboration	Understanding of roles and responsibilities	Factors enabling or hindering collaboration
1. Antenatal care services, including maternal IFA supplementation (75.4% of women with children aged 0–5.9 months received ≥4 ANC visits)	**Clear understanding of roles among FLWs; led by health workers.** “*ASHA will know first who is pregnant in her area because she is always visiting her area. After that*, *she will inform the ANM to provide service*.” (Block health program manager). As part of ANC, AWW registers and weighs women. ANM leads checkups and provides vaccines. ASHA and ANM together provide IFA supplementation.	Most ANC services are provided at VHND, which is the main platform for collaboration among all FLWs.	**Clear roles among FLWs; led by ASHA.** ASHA convenes all the women, provides counseling, and conducts home-based care. AWW registers and weighs women. ANM conducts checkups. ASHA provides IFA supplementation, with ANM and AWW support.	FLWs know each other’s responsibilities, fill in for each other, and closely communicate about any problems. ANC during VHND, providing platform for integrated service delivery.	**Clear roles among FLWs; led by health workers. **ASHA is responsible for calling the beneficiaries. AWW registers pregnant women. ANM is responsible for checkups and providing vaccines and IFA, with support from ASHA.	FLWs fill in for each other, even across sectors. VHND provides the platform for integrated service delivery.
2. Immunization and vitamin A supplementation (69.6% of children aged 12–23.9 months received vitamin A)	**Clear roles among FLWs; led by ANM.** All FLWs involved in preparing beneficiary list; ASHA calls beneficiaries for immunization. ANM administers immunization and vitamin A with ASHA and AWW support.	All FLWs plan and attend monthly immunization sessions together. Guidelines outlining ASHA and AWW responsibilities exist.	**Clear roles among FLWs; led by ANM.** “*The aim of ICDS is to reduce malnutrition and infant mortality. The health department also has the same objective. 100% immunization is the target of ICDS and health department. So*, *we have coordination to achieve this common target*” (Lady supervisor). All FLWs maintain registry and discuss immunization plans a day prior by phone. ASHA and AWW usually mobilize beneficiaries. ANM administers vaccines and vitamin A with ASHA and AWW support.	Immunization involves joint action planning between Health and ICDS. FLWs jointly monitor immunization. FLWs fill in for one another when needed and also coordinate with GKS.	**Clear roles among FLWs; led by ANM**. ASHA is primarily responsible for calling beneficiaries to immunization, usually at AWC. ANM is responsible for immunization and vitamin A, with support.	Planning, implementation, and monitoring of immunization involve all FLWs. Lack of training of ICDS staff or the absence of ANM hinders implementation.
3. Pediatric IFA supplementation (5% of children aged 6–23.9 months received IFA)	**AWW responsible.** AWW primarily provides IFA syrup at AWC.	AWW responsible to provide pediatric IFA, but stocks irregular.	**Roles varied across villages.** ASHA and AWW receive stocks of IFA syrup from ANM to administer, often during VHND. Some AWWs provide IFA at AWC.	IFA is always supplied by Health, but distributed by different FLWs. Unclear lead or primarily responsible.	**Roles varied across villages.** ANM provides IFA syrup with support of ASHA and AWW, or AWW provides IFA at AWC.	IFA is always supplied by Health, but distributed by different FLWs. Unclear lead or primarily responsible.
4. IYCF counseling/education (32.1% of women with children aged 6–23.9 months received CF information during home visits in last 3 months)	**Roles varied across villages. **AWW always present for home visits, with either ANM or ASHA, to provide counseling. At VHND, ANM or ASHA counsels.	While all FLWs involved, ASHA specifically trained for IYCF counseling. Conflicting reports on whether guidelines for home visits exist.	**AWW leads home visits; ANM leads at VHND.** FLWs coordinate via phone regarding home visits, but AWW conducts home visits most often, with ANM and ASHA support. ANM seen as lead for counseling during VHND, although all FLWs involved.	While all FLWs received IYCF training, AWW considered as not qualified to counsel on her own by health workers. Home visits are often missed due to lack of time.	**ANM responsible, but roles varied across villages.** FLWs coordinate home visits, but varied on who conducts. ANM plays lead role in IYCF counseling.	All FLWs reportedly received IYCF training. FLWs coordinate home visits for IYCF counseling.
5. Growth monitoring and referrals for severe acute malnutrition (44.6% of children aged 0–5.9 months, and 51.5% aged 6–23.9 months received growth monitoring)	**AWW leads with support from other FLWs.** AWW is responsible for weighing and plotting growth charts, mainly during VHND. ASHA and ANM support with counseling about growth. At VHND, AWW weighs and measures and, together with ANM, provides referrals. ASHA usually accompanies referred children to hospital.	VHND is a key platform for coordination.	**AWW responsible, often alone.** AWW responsible for weighing and preparing charts, mostly during VHND. ASHA or ANM helps in few villages. AWW fills out referral slips. PRI, SHG, and monitoring committee sometimes help AWW with referrals. ASHA or AWW accompanies referred children.	AWW feels overburdened by growth monitoring large numbers of children alone and asks for more support from ASHA and ANM in this activity.	**AWW leads with support from other FLWs.** AWW responsible with support from ASHA and ANM. AWW follows up with growth monitoring during home visits if absent on VHND. At VHND, AWW weighs children, and ANM makes referrals. ASHA accompanies child to Nutrition Rehabilitation Center, sometimes accompanied by AWW.	FLWs fill in for each other. VHND is key platform for coordination. Guideline for referrals exists.

^a^Mean exposure of interventions across the three districts; little differences existed among the districts [23]

The boldface entries are summary statements, summarizing the longer subsequent text

The VHND provides a common platform for FLWs to work together in delivering the ICDS and health services such as ANC, referrals, growth monitoring, and counseling. One AWW stated, “O*n VHND*, *ASHA calls all the children*, *ANM does checkup of the pregnant ladies*, *and I weigh young girls and women. We give IFA syrup to children. ANM worker checks the health of the 3 to 5 years old children*”. In all three districts, coordination among FLWs varied depending on the type of services delivered. There was a clear understanding of roles and responsibilities and good coordination among the FLWs for implementing ANC, immunization and vitamin A supplementation. One ANM stated, “*My duty is to check the pregnant women and advise them about their diet and give them IFA and TT* [*tetanous toxoid*]. “*If the pregnant woman does not come (to VHND) with her child*, *then the ASHA worker goes to call and brings her*.” In parallel with these findings, exposure to the interventions usually provided during VHND, particularly ANC, immunization and vitamin A supplementation, and growth monitoring across the three districts were generally over 50% [23] (Table 3).

On the contrary, FLWs across the three districts demonstrated varying levels of understanding of their roles in providing IYCF counseling and pediatric IFA supplementation. For instance, in District 2, one ANM stated that AWW, ANM and ASHA plan together for counseling over the phone and jointly conduct home visits. In District 1, an ASHA informed that AWW accompanies her to home visits whenever she calls her, and ANM also comes along only when it is required, such as in cases of an underweight child. In District 3, one ANM stated that she plans for home visits with AWW, and another ANM said that she coordinates the action plan for home visits with ASHA and AWW. While there appears to be overall coordination to deliver the service, there is ambiguity on who leads the counseling and how it is organized. Similarly in the patterns of exposure, less than one-third of beneficiaries had been exposed to these two interventions across the three districts [23] (Table 3).

In general, there is a common understanding among the FLWs that they need each other to effectively deliver services. One AWW said, “*All three of us- ANM*, *ASHA*, *and I work together. We all decide before doing anything*”, and further explained, “*We have good coordination; ASHA is always with us. We respect each other*’*s work and discussion*.” However, the lack of time or heavy workload, scheduling conflicts, and poor communication were named as barriers to coordination. AWWs and ANMs perceive that VHND increased their workload. In addition, after the introduction of the cadre of ASHAs at the village level, some block-level officials observed that AWWs are less motivated to deliver services for which ASHAs are receiving incentives. For example, AWWs usually weigh the children for monthly growth monitoring, but ASHAs receive incentives specifically for referring children (based on the growth monitoring results) to the nutrition rehabilitation center; thus, AWWs become discontented by the unbalanced incentive system.

## Discussion

Intersectoral convergence may be considered as a process towards achieving higher efficiency, quality, coverage, and effectiveness or as an end in itself, i.e. holistic approach to public health and nutrition. In our study, we focus on the conditions of convergence as a process in service delivery, but with the ultimate vision of a holistic approach to improve maternal and child health and nutrition. Effective convergence between the health and nutrition sectors relies on various factors for improved intersectoral actions and positive outcomes. The findings of our study highlight the existence of a mandate for convergence for health and nutrition in the form of policy and guidelines at the state level, understood and articulated by the leadership in both sectors. There is a shared understanding of the goals and priority actions. In practice, however, we find that there is limited joint planning and coordination due to demands arising from the core sectoral priorities. The district is intended to be the site of planning and capacity building; the block is the site of training, supervision and support, (joint review meetings, supervisory field visits, training, and periodic data review); and village includes the site of service delivery, which is the domain of the FLWs. Our results indicate that the nature and extent of coordination at the district, block, and village levels varied and were service-specific, with largest gaps in coordination appearing at the block level. Heavy workloads, narrow accountability to sectoral outcomes, and limited supervisory mechanisms were common challenges to coordination across all levels. Our findings concur with results from another field study of various sites in India that highlighted needs for nutrition-focused outreach to families and more structured collaboration between health and nutrition [27].

Historically, the DHFW and DWCD worked toward a common goal to reduce infant mortality, which facilitated coordinated action and effective program implementation [28]. At the state level, several joint guidelines and program reviews have been implemented around this common goal. However, the sectoral domination of the health department is perceptible in our results. This is likely because several of the essential interventions to reduce infant mortality such as immunization are delivered by the health department. In addition, the focus on reducing infant mortality and treatment of severely malnourished children during the last two decades has potentially diverted action from strengthening preventive actions such as counseling for IYCF and prevention of illness [28], thus lending salience to the activities of the health department. Indeed, in our analysis, we find that the district-level and block-level coordination meetings are predominantly driven by reviewing of the health indicators and tallying of numbers rather than a comprehensive feedback or review of programmatic operations. Although NRHM has a broad objective of strengthening the primary healthcare system, it focuses its efforts on infant and maternal mortality, thus coordination meetings are dominated by a numerical review of their indicators [29]. This narrow focus of priorities and processes, however, is unlikely to result in effective and meaningful improvements coordination or service delivery for nutrition interventions like counseling.

Frontline worker coordination, therefore, appears to be good for services that are primarily driven by the health department (e.g., ANC and immunization) than for services that require more joint planning such as counseling for IYCF. Coordinated functioning is a result of understanding among FLWs of their tasks, guided by protocols, tools and instruments; these are well defined for most of the health services. Similarly, for VHND, where several health and nutrition services are provided, there is a protocol and clear guidelines for FLW roles. Although guidelines for IYCF counseling exist, FLWs are not fully aware of them. Furthermore, services such as immunization have been delivered by the health system for much longer time than the more recent IYCF counseling services, which could contribute to better coordination among the FLWs in the delivery of health services compared to other services.

In our study, FLWs valued working together and realized their interdependent roles in delivering the services. However, inadequate or unbalanced incentives, differences in training (e.g., ANM is a trained paramedic while ASHA and AWW are not) and work roles (e.g., ANM vaccinates children, while AWW and ASHA mobilize them) may lead to resentment [29]. Mutual respect, support and understanding of their own responsibilities is critical for FLWs to work together, and this can be facilitated from the district and block levels through issuing of clear guidelines and ensuring that each of the FLW’s work is given adequate recognition and prominence.

Overall, service-specific coordination, for example, coordination focused solely on mobilizing mothers for immunization services, hinders the vision of achieving a holistic approach. An integrated approach to ensuring the delivery of all the essential services to the mother-child dyad during the first 1000 days is needed to avoid the current service-specific coordination that prioritizes certain services over others. While the sectoral focus is important to deliver on the outcomes for specific services, it can dilute the efforts to ensure coverage of all the required services for the mother-child dyads. Thus, for example, review meetings focusing on immunization and vitamin A supplementation may be strengthened by including the review of food supplement distribution and counseling, both of which are also often targeted to the same eligible mothers and children. It is also imperative that leadership at higher levels ensures that the two departments are held accountable for planned actions and delivering of all the services using the continuum of care approach.

There are a few limitations to our study. First, the study was conducted in purposively selected districts; therefore, the results are not representative of the entire state. However, our sampling was performed to draw from different districts to maximize the variations in service delivery context within the state, and the qualitative data were collected from all key actors at the different administrative levels involved with the health and ICDS programs. Our study is based on single cross-sectional interviews, and we do not claim causal relationships between the degrees and outcomes of convergence, apart from linkages stated directly by interview respondents. We analyzed iteratively for themes and patterns of influencing factors and linked consequences that emerged from the qualitative data, in order to examine potential processes and relationships. While responses were based on recall, which is vulnerable to bias, we triangulated findings from various respondents.

Our study findings contribute to the growing evidence on multisectoral convergence processes and hold relevance for other countries committed to scaling up nutrition interventions, where coordination has been identified as a major challenge [30]. We applied a comprehensive approach to examine the degrees and conditions of intersectoral convergence at the different administrative and operational levels of the health and nutrition systems and to trace implementation of specific interventions, and this systematic approach may be useful in other settings to identify gaps and elements needed for strengthening coordination.

## Conclusions

Delivery of health and nutrition services operate on the assumption that intersectoral coordination is important and will take place accordingly based on common objectives/purposes, but multiple factors influence on convergence decision and action points. Also, different degrees and conditions of convergence are in play among actors at different operational levels. This may be reflective of the varying administrative and operational functions at the different levels, thereby uniformity of relations may not be feasible nor necessary. However, across these forms of interrelations, there is a need for congruence or shared priorities in actions, clear roles and leadership, and regularity of participation and actions across all levels, as well as sectoral accountability for convergence. As convergence is a means to achieving effective coverage and delivery of services for improved maternal and child health and nutrition, focus should also be on delivering all the essential services to the mother-child dyads through mechanisms that facilitate a continuum of care approach, rather than sectorally-driven, service-specific delivery processes.

## Additional files

Additional file 1: State-level Interview Guide: Guide used for interviewing district-state officials from Health and ICDS as well as development partners. The interview guide includes questions about work responsibilities and convergence at the state, district and block levels. (PDF 508 kb)
Additional file 2: District-level Interview Guide: Guide used for interviewing district-level officials from Health and ICDS such as the district program manager, district community mobilizer, and district project officer. The interview guide includes questions about work responsibilities, coordination of Health and ICDS services, and specific mechanisms of convergence at the district level. (PDF 642 kb)
Additional file 3: Block-level Interview Guide: Guide used for interviewing block-level officials from Health and ICDS such as the block program manager, block community mobilizer, and child development project officer. The interview guide includes questions about work responsibilities, coordination of Health and ICDS services, specific mechanisms of convergence, VHND, supervision and training, and work support at the block level. (PDF 686 kb)
Additional file 4: Frontline Worker Interview Guide: Guide used for interviewing frontline workers from Health and ICDS, particularly the ANM, ASHA, and AWW. The interview guide includes questions about workload, coordination of Health and ICDS services, specific mechanisms of convergence, VHND, supervision, and job motivation and satisfaction at the FLW level. (PDF 679 kb)

## Abbreviations

ANC: antenatal care
ANM: Auxiliary Nurse Midwife
ASHA: Accredited Social Health Activist
AWC: Anganwadi Center
AWW: Anganwadi Worker
DHFW: Department of Health and Family Welfare
DWCD: Department of Women and Child Development
FLW: Frontline worker
ICDS: Integrated Child Development Services
IFA: Iron and folic acid
IYCF: Infant and young child feeding
MoHFW: Ministry of Health and Family Welfare
MoWCD: Ministry of Women and Child Development
NRHM: National Rural Health Mission
VHND: Village Health and Nutrition Days
