# Supplementary material for: Understanding the role of intersectoral convergence in the delivery of essential maternal and child nutrition interventions in Odisha, India: a qualitative study
Source: BMC Public Health. 2017 Feb 2;17:161. doi: 10.1186/s12889-017-4088-z (PMC5290617; doi:10.1186/s12889-017-4088-z)
Supplement: Additional file 1: — State-level Interview Guide: Guide used for interviewing district-state officials from Health and ICDS as well as development partners. The interview guide includes questions about work responsibilities and convergence at the state, district and block levels. (PDF 508 kb) [file 12889_2017_4088_MOESM1_ESM.pdf]

STATE-LEVEL INTERVIEW GUIDE

Types of respondents: ICDS head; Health dept. head; Directorate ICDS; Directorate NRHM; ICDS Training institute head; NRHM Training Institute head; Public health directorate; and development partners

INFORMED CONSENT* - Obtain permission from the interviewee to be interviewed.

Proceed after the interviewee gives consent to be interviewed:

INTERVIEWER FILL OUT AT BEGINNING OF INTERVIEW

1.	Time interview started	☐☐Hrs ☐☐Min

INTERVIEWER FILL OUT AT THE END OF INTERVIEW

2.	Time interview ended	☐☐ Hrs ☐☐ Min
3.	Location of the interview	________________
4.	Which language did the respondent speak? (circle the response)	1. Hindi<br>2. Odiya<br>-87 Other (specify) ________________
5.	Who was present at the interview? *(List all the people including the interviewer and the note taker)*	
6.	What is your assessment of the respondent's accuracy?	1. Good / okay<br>2. Not so good / bad (provide comments)
**Interviewer Comments**		

**Interview Status (Circle the appropriate choice)**	
**Completed**	**Respondent unavailable**
**Not found**	**Respondent temporarily unavailable**
**Refused part way**	**Refused full interview**
**Recorded** (Yes) (No)	

Work responsibilities:

1. Since how long have you been in your current position? What was your posting before this?

2. What are your main responsibilities in your current position?

3. How has been your experience in working in your current position?

[Probe for]

- Regular functions/roles/responsibilities of the respondent
- Perceptions about specific systems/government personnel/incumbents
- Changes over time in experience/interactions

Convergence among state-level actors:

4. What do you think are the major issues concerning maternal and child nutrition in Odisha/MP? *[e.g., MCN problems, operational issues (e.g. resources, delivery, coverage, etc.), sociopolitical issues, etc].* What actions are being taken to address these issues?

5. Which state departments/individuals play a role in these actions to improve maternal and child nutrition in Odisha/MP? How are they involved? *[focusing on direct interventions]*

[Probe for]

- Perceived roles of ICDS/NRHM
- Roles of any other departments

6. ***Ask Q6 only if it is a development partner*****:** What is your organization's strategy and role regarding health and nutrition in this state?

7. Which of the departments/individuals do ***you*** work closely with in any of these actions Odisha/MP? In what ways do you work together? *[focusing on direct interventions]*?

8. What do you understand by "convergence"?

9. What are your thoughts about the need for convergence between health and nutrition is necessary? Why/in what ways?

[Probe for]

- Instances/examples of convergence successfully changing outcomes/hindering progress towards positive outcomes

10. How is convergence between health and nutrition planned and implemented in the state?

[Probe for]

- National guidelines
- APIP shared objectives
- Donor partner programmes
- Formulation of district level guidelines

11. What are some of the ways in which convergence between health and nutrition has been brought about/implemented at the state level? What is your opinion about these mechanisms?

[Probe for]

- Budgetary allocations
- Organization of line departments (Do implementation plans of various line departments adequately reflect the actions for convergence that are outlined in policy documents?)
- Policy guidelines/task forces
- Political will
- Operational guidelines
- Have common frameworks for monitoring been developed that have buy-in and ownership of convergent departments?
- What types of indicators are in place to measure nutrition-linked outcomes between both sectors?
- What kind of processes for feedback and accountability are set up between the two departments?
- What kind of monitoring indicators are available to assess convergent actions?
- Initiatives to build capacity within your department areas and for convergent actions
- Initiatives by the government/civil society/donor partners
- Coordinated planning/training/implementation/monitoring

12. What are/have been the main facilitators and barriers to convergence at the state level? How can/were they overcome (solutions)?

[Probe for]

- Budgetary allocations
- Organization of line departments
- Current incumbents
- Policy guidelines/task forces
- Political will
- Operational guidelines
- Initiatives by the government/civil society/donor partners
- Coordinated planning/training/implementation/monitoring
- Gaps in fulfilling respective roles and perceived reasons
- Lack of convergence and perceived reasons
- Potential for new actors/arrangements

13. Who/which departments should be the main actors in these solutions? What are their potential roles?

[Probe for]

- Roles of main actors (politicians/bureaucrats/civil society/NRHM/ICDS/funding agencies/private sector

14. Please tell me at which of the levels (i.e., *state, district, block, and village-levels)* do you think that coordination is most needed? In what ways?

Convergence at the district and local levels:

15. What mechanisms of convergence between health and nutrition are in place/necessary at the district, block, and village-levels? *[focusing on direct interventions]*

16. How are these mechanisms of convergence at the district, block, and village-levels monitored?
