# Supplementary material for: Understanding the role of intersectoral convergence in the delivery of essential maternal and child nutrition interventions in Odisha, India: a qualitative study
Source: BMC Public Health. 2017 Feb 2;17:161. doi: 10.1186/s12889-017-4088-z (PMC5290617; doi:10.1186/s12889-017-4088-z)
Supplement: Additional file 2: — District-level Interview Guide: Guide used for interviewing district-level officials from Health and ICDS such as the district program manager, district community mobilizer, and district project officer. The interview guide includes questions about work responsibilities, coordination of Health and ICDS services, and specific mechanisms of convergence at the district level. (PDF 642 kb) [file 12889_2017_4088_MOESM2_ESM.pdf]

DISTRICT-LEVEL INTERVIEW GUIDE

Types of Respondents:
ICDS - District Project Officer (DPO)
Health - District Program Manager (DPM), District Community Mobilizer (DCM)

INTERVIEWER FILL OUT BEFORE LEAVING FOR INTERVIEW

**Place**		**Respondent**			
State (State code)		Respondent's name			
		Respondent job title			
District (District code)		Respondent department			
		Date of the interview	(dd)	(mm)	(yyyy)
**FILE NAME :**					

INFORMED CONSENT* - *Obtain permission from the interviewee to be interviewed.

Proceed after the interviewee gives consent to be interviewed:

INTERVIEWER FILL OUT AT BEGINNING OF INTERVIEW

1.	Time interview started	☐☐Hrs ☐☐Min

INTERVIEWER FILL OUT AT THE END OF INTERVIEW

2.	Time interview ended	☐☐ Hrs ☐☐ Min
3.	Location of the interview	1. District office<br>2. -87 Other (Specify) ________________
4.	Which language did the respondent speak? (circle the response)	1. Hindi<br>2. Odiya<br>-87 Other (specify) ________________
5.	Who was present at the interview? *(List all the people including the interviewer and the note taker)*	
6.	What is your assessment of the respondent's accuracy?	1. Good / okay<br>2. Not so good / bad (provide comments)
**Interviewer Comments**		

**Interview Status (Circle the appropriate choice)**	
**Completed**	**Respondent unavailable**
**Not found in the block**	**Respondent temporarily unavailable**
**Refused part way**	**Refused full interview**
**Recorded** (Yes) (No)	

**FOR SUPERVISOR ONLY**	**Date (dd/mm/yyyy)**					**Name of the supervisor**
Supervisor reviewed the interview						

Instructions: ***Where multiple choices exist (ICDS/Health), CIRCLE the appropriate type of respondent you are directing the questions.***

[italics] **= instructions to interviewer**
(italics) **= examples or concepts for the interviewer to keep in mind.**

A. GENERAL INFORMATION

1. How long have you been working as _____________?

B. WORK RESPONSIBLITIES AND WORKLOAD

2. What are your main responsibilities as ___________?

3. Who do you supervise? *[Interviewer: type of people that report to them]*

C. ICDS AND HEALTH SERVICES AND COORDINATION

4. What services and programs do you provide for mothers and children <2 years?

   [If not mentioned, probe for:]
   1-Breastfeeding counseling
   2-Counseling on complementary feeding, including food safety, hygiene and sanitation
   3-Maternal care and nutrition (for pregnant and lactating women)
   4-IFA supplementation for 6-36 months and reproductive-age women
   5-Immunization
   6-Vitamin A supplementation
   7-Deworming
   8-Diarrhea treatment and prevention (ORS)
   9-SAM treatment
   10-Malaria treatment and prevention (bed nets)
   11-Growth monitoring
   12-Antenatal care
   13-Supplementary nutrition for mothers and children
   14-Any other programs specific to the district

5. Do you provide home visits?

   [Probe:]
   - Services covered

6. Which of the services require close coordination with (ICDS/Health) at the district-level?

7. Among these, where do you have **good** coordination with (ICDS/Health) at the district-level?

   [Probe:]

- What makes you say that it is good? *(definition of good coordination)*
- Tell me about how you coordinate.
- What helps with coordination? *(facilitators to coordination)*
- What makes it difficult to coordinate? *(barriers to coordination)*

8. In which service have you had **poor** coordination with (ICDS/Health) at the district-level?

[Probe:]
- What makes you say that it is poor? *(definition of poor coordination)*
- Tell me about what happened.
- Was there coordination for this service before?
- What has made it difficult to coordinate now? *(barriers to coordination)*
- Did you try (doing) anything to make the situation better?
- Other instances where you faced poor coordination?

[If no or minimum coordination:]
- Do you think there should be more coordination?
- What is keeping you from having more coordination?
- What would be helpful in making this possible?

D. SPECIFIC MECHANISMS OF CONVERGENCE

9. Please tell us how do you coordinate action plans with (ICDS/Health) at the district-level?

[Probe:]
-
- What do the action plans usually include?
- Anything that helps/makes it difficult to prepare the plans? *(facilitators and barriers)*
- Any state guidelines on how to prepare joint action plans?

10. Tell us how do you coordinate any trainings with (ICDS/Health) at the district-level?

[Probe:]
- What do the trainings usually cover? *(type/purpose of training)*
- Who receive these trainings?
- How often do you coordinate the trainings?
- Anything that helps/makes it difficult to coordinate? *(facilitators and barriers)*
- What would be helpful to make the coordination better?
- Any guidelines from the state on how to coordinate joint training?

[If no coordination:]
- Do you think there should be coordination for trainings?
- What would be helpful in making this possible?

11. Tell us how do you coordinate sector meetings with (ICDS/Health) at the district-level?

[Probe:]

- What is usually covered during the meetings?
- What happens after the meetings? *[e.g., follow-up, actions]*
- How often do you coordinate the meetings?
- Anything that helps/makes it difficult to coordinate? *(facilitators and barriers)*
- What would be helpful to make the coordination better?

[If no coordination:]

- Do you think there should be joint meetings?
- What would be helpful in making this possible?

12. Tell us how do you coordinate <u>supervision visits</u> with (ICDS/Health) at the district-level?

[Probe:]

- With whom do you usually conduct supervision visits?
- For what purpose do you usually coordinate supervision visits?
- How often do you coordinate the visits?
- Anything that helps/makes it difficult to coordinate? *(facilitators and barriers)*
- What would be helpful to make the coordination better?
- Any guidelines from the state on how to coordinate supervision?

[If no coordination:]

- Do you think there should be coordination for supervision?
- What would be helpful in making this possible?

13. Besides meetings, trainings and supervision, do you coordinate with (ICDS/Health) in anything else?

14. Please tell us how coordination among the district officials influences service delivery?

15. What are your thoughts about how the overall coordination with (ICDS/Health) is working? *(e.g. helpful, not always practical or feasible, time consuming, not needed)*

[Probe:]

- Do you have any suggestions on ways to improve coordination?

16. Please tell us about how guidelines are provided to block officials regarding coordination with (ICDS/Health)

[Probe:]

- Any guidelines for developing block micro plans?
- Any guidelines for coordinating trainings?
- Any guidelines for coordinating supervision?
- Any guidelines for coordinating sector meetings?
- Any guidelines for coordinating VHNDs?

DEMOGRAPHIC INFORMATION

Lastly, I would like to ask you some information about your background.

17. Could you please tell me your age?	About ___________ years<br>88. Don't know/Do not want to tell
18. What is the highest level of schooling you have completed and passed?<br><br>*[Count only up to the level <u>passed</u>.]*	1. Did not complete Class 1<br>2. Class 1<br>3. Class 2<br>4. Class 3<br>5. Class 4<br>6. Class 5<br>7. Class 6<br>8. Class 7<br>9. Class 8<br>10. Class 9<br>11. Class 10<br>12. Class 11<br>13. Class 12<br>14. Graduate and above<br>15. Other diploma<br>16. Madrassa (if no formal classes exist)<br>17. Tuitions / teaching at home<br>18. Other training / courses / camps only<br>19. No schooling<br>87. Other (specify)_______________

Thank you!
