# Supplementary material for: Understanding the role of intersectoral convergence in the delivery of essential maternal and child nutrition interventions in Odisha, India: a qualitative study
Source: BMC Public Health. 2017 Feb 2;17:161. doi: 10.1186/s12889-017-4088-z (PMC5290617; doi:10.1186/s12889-017-4088-z)
Supplement: Additional file 3: — Block-level Interview Guide: Guide used for interviewing block-level officials from Health and ICDS such as the block program manager, block community mobilizer, and child development project officer. The interview guide includes questions about work responsibilities, coordination of Health and ICDS services, specific mechanisms of convergence, VHND, supervision and training, and work support at the block level. (PDF 686 kb) [file 12889_2017_4088_MOESM3_ESM.pdf]

BLOCK-LEVEL INTERVIEW GUIDE

Types of Respondents:
ICDS - Child Development Project Officer (CDPO), Lady Supervisors (LS)
Health - Block Program Manager (BPM), Public Health Extension Officer (PHEO)/Block Community Mobilizer (BCM)

INTERVIEWER FILL OUT BEFORE LEAVING FOR INTERVIEW

**Place**		**Respondent**			
State (State code)		Respondent's name			
		Respondent job title			
District (District code)		Respondent department			
		Date of the interview	(dd)	(mm)	(yyyy)
Block (Block code)					
**FILE NAME :**					

***INFORMED CONSENT* - Obtain permission from the interviewee to be interviewed.**

Proceed after the interviewee gives consent to be interviewed:

INTERVIEWER FILL OUT AT BEGINNING OF INTERVIEW

1.	Time interview started	☐☐Hrs ☐☐Min

INTERVIEWER FILL OUT AT THE END OF INTERVIEW

2.	Time interview ended	☐☐ Hrs ☐☐ Min
3.	Location of the interview	1. Block office<br>2. HSC<br>3. -87 Other (Specify) _______________
4.	Which language did the respondent speak? (circle the response)	1. Hindi<br>2. Odiya<br>-87 Other (specify) _______________
5.	Who was present at the interview? *(List all the people including the interviewer and the note taker)*	
6.	What is your assessment of the respondent's accuracy?	1. Good / okay<br>2. Not so good / bad (provide comments)
**Interviewer Comments**		

**Interview Status (Circle)**	
**Completed**	**Respondent unavailable**
**Not found in the block**	**Respondent temporarily unavailable**
**Refused part way**	**Refused full interview**
**Recorded** (Yes) (No)	

**FOR SUPERVISOR ONLY**	**Date (dd/mm/yyyy)**					**Name of the supervisor**
Supervisor reviewed the interview						

Instructions: ***Where multiple choices exist (ICDS/Health), CIRCLE the appropriate type of respondent you are directing the questions.***

[italics] **= instructions to interviewer**
(italics) **= examples or concepts for the interviewer to keep in mind**

A. GENERAL INFORMATION

1. How long have you been working as _____________?

2. What was your previous job/position?

B. WORK RESPONSIBLITIES AND WORKLOAD

3. What are your main responsibilities as ___________?

4. Who do you supervise?

   [Probe:]
   - How many?

C. ICDS AND HEALTH SERVICES AND COORDINATION

5. What services for mothers and children <2 years do you provide?

   [If not mentioned, probe for:]
   1-Breastfeeding counseling
   2-Counseling on complementary feeding, including food safety, hygiene and sanitation
   3-Maternal care and nutrition (for pregnant and lactating women)
   4-IFA supplementation for 6-36 months and reproductive-age women
   5-Immunization
   6-Vitamin A supplementation
   7-Deworming
   8-Diarrhea treatment and prevention (ORS)
   9-SAM treatment
   10-Malaria treatment and prevention (bed nets)
   11-Growth monitoring
   12-Antenatal care
   13-Supplementary nutrition for mothers and children

6. How is _____(service)________ provided?

   [Probe:]
   - Who provides?
   - Ways (ICDS/Health) is involved
   - How coordinate with (ICDS/Health)

If not mentioned, then probe:

- Where are services such as counseling on infant and young child feeding, immunization, and pregnancy care provided?
- Who provides them?
- How do the different workers coordinate home visits?

7. Which of these services require close coordination with (ICDS/Health) among block-level officials?

8. Among these, where do you have **good** coordination with (ICDS/Health) at the block-level?

   [Probe:]
   - What makes you say that it is good? *(definition of good coordination)*
   - Tell me about how you coordinate, from planning to service delivery.
   - What helps you to coordinate? *(facilitators to coordination)*
   - What makes it difficult to coordinate? *(barriers to coordination)*

9. In which service have you had **poor** coordination with (ICDS/Health) at the block-level?

   [Probe:]
   - What makes you say that it is poor? *(definition of poor coordination)*
   - Tell me about what happened.
   - Was there coordination for this service before?
   - What has made it difficult to coordinate now? *(barriers to coordination)*
   - Did you try (doing) anything to make the situation better?
   - Are there other instances where you faced poor coordination?

   If there is no poor coordination, then:
   - In which service do you think there needs to be more or better coordination?
   - How could this coordination be improved?

D. SPECIFIC MECHANISMS OF CONVERGENCE

10. Please tell me about how you coordinate any trainings with block officials in (ICDS/Health)?

    [Probe:]
    - What do the trainings usually cover? *(type/purpose of training)*
    - How often do you coordinate the trainings?
    - Anything that helps/makes it difficult to coordinate? *(facilitators and barriers)*
    - What would be helpful to make the coordination better?
    - Any guidelines on how to coordinate trainings?

    [If no coordination:]
    - Do you think there should be coordination for trainings?
    - What would be helpful in making this possible?

11. Tell me how you coordinate supervision visits with block officials in (ICDS/Health)?<br><br>*[Probe:]*<br>• With whom do you usually conduct supervision visits?<br>• For what purpose do you most often coordinate supervision?<br>• How often do you coordinate the visits?<br>• Anything that helps/makes it difficult to coordinate? *(facilitators and barriers)*<br>• What would be helpful to make the coordination better?<br>• Any guidelines on how to coordinate supervision?<br><br>*[If no coordination:]*<br>• Do you think there should be coordination for supervision?<br>• What would be helpful in making this possible?
12. Tell me how you coordinate sector meetings with block officials in (ICDS/Health)?<br><br>*[Probe:]*<br>• What is usually covered during the meetings?<br>• How often do you coordinate the meetings?<br>• Anything that helps/makes it difficult to coordinate? *(facilitators and barriers)*<br>• What would be helpful to make the coordination better?<br>• Any guidelines on how to coordinate sector meetings?<br><br>*[If no coordination:]*<br>• Do you think there should be coordination for sector meetings?<br>• What would be helpful in making this possible?
13. Besides meetings, trainings and supervision, do you coordinate with block officials of (ICDS/Health) in anything else? *[e.g., developing action plans]*
14. *[*Insert Question about any district-specific mechanisms of coordination, if any identified during district interviews.]*
15. What is the role of Village Health Sanitation Committees (VHSCs) in promoting services?
16. What role do VHNSCs play in promoting coordination between ICDS and Health?
17. Please tell me how is PRI involved in promoting maternal and child nutrition?<br><br>*[Probe:]*<br>• Role of PRI members in promoting coordination between ICDS and Health?

18. How do you think coordination with (ICDS/Health) at the block-level is influencing service delivery? *(e.g. more efficient, increase coverage, or no changes)*

19. What are your thoughts about how the overall coordination with (ICDS/Health) is working? *(e.g. helpful, not always practical or feasible, time consuming, not needed)*

    [Probe:]
    - Do you have any suggestions on ways to improve coordination?

E. ROUTINIZATION OF VILLAGE HEALTH AND NUTRITION DAYS (VHND)

20. Tell me how VHNDs started in your area?

    [Probe:]
    - When did it start?
    - Reason for starting VHND. Was there someone or some policy/guidelines that initiated it?

21. Please tell me about how VHNDs are organized in your block.

    [Probe:]
    - How often are VHNDs conducted?
    - Where are they usually conducted?
    - Any special order/way in how activities are conducted?
    - Any rituals established with VHNDs? *(e.g. ceremonies, celebrations, periodic gatherings)*
    - Reasons for conducting these rituals?
    - Any guidelines or manual used?

22. Tell me about the staff involved in planning and conducting VHNDs in your block. *(i.e., block officials and FLWs)*

    [Probe:]
    - How do they know how to conduct VHNDs? *(e.g. training or orientation on conducting VHNDs)*
    - How often is there a shortage of staff for conducting VHNDs? How do you handle it?

23. How did <u>you</u> learn about how to organize and conduct VHNDs?

    [Probe:]
    - Training/orientation received? *(what was learned)*
    - Who provided the training/orientation?
    - Other participants?

24. What services are usually provided during the VHNDs?

[If not mentioned, probe for:]

1-Breastfeeding counseling
2-Counseling or community discussions on complementary feeding, including food safety, hygiene and sanitation
3-Counseling on maternal care and nutrition (for pregnant and lactating women)
4-IFA supplementation for 6-36 months and reproductive-age women
5-Immunization
6-Vitamin A supplementation
7-Deworming
8-Diarrhea treatment and prevention (ORS)
9-SAM referrals
10-Malaria treatment and prevention (bed nets)
11-Growth monitoring
12-Registration of pregnant women
13-Antenatal care
14-Supplementary nutrition provided for underweight children

[Probe:]

- How often are these services provided during VHNDs? *(frequency of service)*
- How much time is spent on providing these activities during VHNDs? How do you handle it?

25. How is money/funds used to conduct VHNDs?

[Probe:]

- Different uses of money in conducting VHNDs? *(e.g. incentives for AWW or ASHA, preparation of complementary food, THR, etc.)*
- Where does the money come from? *(source of money)*
- Is the money usually enough to cover the needs? How do you handle it?
- For how long do you think that these funds will be available?

26. Tell me about the materials or equipment used for VHNDs.

[Probe:]

- Types of materials or equipment are required to conduct VHNDs? *(e.g. weighing scales, table, screen, medical supplies, THR, referral slips, IEC materials for counseling, MCPC cards, vaccinations, IFA tablets, deworming tablets, etc.)*
- Where do they come from? *(source of materials and equipment)*
- Are there usually enough? How do you handle it?
- For how long do you think that the materials or equipment will be available?

27. Who usually participates in VHND?

[Probe:]

- Who attends VHNDs? *(e.g. pregnant or lactating women, children, adolescents, newly married couples, etc.)*
- Which of the ICDS or Health officials attend VHNDs? How often do they attend?

28. How do people recognize VHND or talk about it?

[Probe:]

- Any visible logos, images or pictures that are commonly used to represent VHND?
- Any specific names or words for VHNDs? *[especially if VHND called by a different name]* Any words or phrases commonly used during VHNDs?

29. How are VHNDs usually supervised?

[Probe:]

- Who from ICDS and Health/NRHM supervises VHNDs?
- What is usually done/checked when VHNDs are visited?
- Any documents or checklists used to supervise VHNDs?
- What are the criteria used to decide if a VHND is successful?

30. Tell me about any changes that have been made to the VHNDs since it first started.

[Probe:]

- Types of changes were made for the staff working on VHNDs? *(e.g. VHND task assignment to different staff involved, changes to supervision)*
- Types of changes were made to the facility, materials or equipment for VHNDs? *(e.g. painting of AWCs, new IEC materials, new equipment, etc.)*
- Any changes made to the types of activities conducted at VHNDs?
- Any changes made to how activities are organized or conducted?
- Reasons for making these changes? *[Probe for each of the changes.]*

[If no changes made:]

- What are some changes you would like to make to VHNDs?
- What keeps/stops you from making any changes?

31. In which ways do you think VHND is helping or not helping in delivering the services??

[Probe:]

- Ways service delivery improved because of VHND? *(e.g. quality, organization, coverage)*
- Ways service delivery worsened because of VHND?
- Any influence VHNDs have on the work of your staff? *(e.g., make the work more difficult, help improved the work, etc.)*
- Any quality of services changes?

[If no changes:]

- Reasons for no changes?
- What needs to be done to make improvements?

32. How do you think VHND is influencing utilization of services?

[Probe:]

- Ways service utilization activities improved because of VHND? *(e.g. coverage, access, timeliness)*
- What helped to make such improvements?
- Ways service utilization worsened because of VHND?
- What difficulties led to such deterioration?

[If no changes:]

- Reasons for no changes?
- What needs to be done to make improvements?

F. WORK SUPERVISION

33. Who is your immediate supervisor?

34. Tell me about the meetings with your supervisor.

[Probe:]

- Purpose of the meetings? *(topics covered)*
- In the last 6 months, how many times did your supervisor visit you?
- Purpose of your supervisor's last visit?
- When do you expect to meet your supervisor again?

G. WORK TRAINING

35. What kinds of training have you received in the past 2 years?

H. JOB SATISFACTION AND WORK SUPPORT

36. How do you feel about working as _____________?

[Probe:]

- What makes you feel this way?
- When do you feel this way*?* *(e.g. specific situations or events)*

37. Tell me about the support you receive from your superiors *(i.e. district-level)*?

[Probe:]

- Which of your superiors provide support to you?
- Types of support do you receive?
- Other types of services that would help you?

[If no support:]

- Types of support you would like to receive?

38. What are your thoughts about the adequacy of staffing in support of your work?<br><br>*[Probe:]*<br>• How often do (LS/AWW/ANM/ASHA) change in your block? *(personnel turnover)*
39. Tell me about the support you receive from the civil society/community or other groups?<br><br>*[Probe:]*<br>• Which civil society groups or organizations actively support you?<br>• Types of support usually provided?<br><br>*[If no support:]*<br>• Reasons for not receiving support?<br>• What types of support would you like to receive?

DEMOGRAPHIC INFORMATION

Lastly, I would like to ask you some information about your background.

40. Could you please tell me your age?	About ___________ years<br>88. Don't know/Do not want to tell
41. What is the highest level of schooling you have completed and passed?<br><br>*[Count only up to the level <u>passed</u>.]*	1. Did not complete Class 1<br>2. Class 1<br>3. Class 2<br>4. Class 3<br>5. Class 4<br>6. Class 5<br>7. Class 6<br>8. Class 7<br>9. Class 8<br>10. Class 9<br>11. Class 10<br>12. Class 11<br>13. Class 12<br>14. Graduate and above<br>15. Other diploma<br>16. Madrassa (if no formal classes exist)<br>17. Tuitions / teaching at home<br>18. Other training / courses / camps only<br>19. No schooling<br>87. Other (specify)_______________

Thank you!
