# Supplementary material for: Understanding the role of intersectoral convergence in the delivery of essential maternal and child nutrition interventions in Odisha, India: a qualitative study
Source: BMC Public Health. 2017 Feb 2;17:161. doi: 10.1186/s12889-017-4088-z (PMC5290617; doi:10.1186/s12889-017-4088-z)
Supplement: Additional file 4: — Frontline Worker Interview Guide: Guide used for interviewing frontline workers from Health and ICDS, particularly the ANM, ASHA, and AWW. The interview guide includes questions about workload, coordination of Health and ICDS services, specific mechanisms of convergence, VHND, supervision, and job motivation and satisfaction at the FLW level. (PDF 679 kb) [file 12889_2017_4088_MOESM4_ESM.pdf]

FRONTLINE WORKER INTERVIEW GUIDE

Types of Respondents:
ICDS - Anganwadi Worker (AWW)
Health - Auxillary Nurse Midwife (ANM)/Health Worker Female (HWF) [or Health Worker Male (HWM)], and Accredited Social Health Activist (ASHA)

INTERVIEWER FILL OUT BEFORE LEAVING FOR INTERVIEW

**Place**		**Respondent**			
State (State code)		Respondent name	Respondent job title		
District (District code)		Respondent department			
Block (Block code)		Date of the interview	(dd)	(mm)	(yyyy)
Village (Village code)					
**FILE NAME :**					

INFORMED CONSENT* - *Obtain permission from the interviewee to be interviewed.

Proceed after the interviewee gives consent to be interviewed:

INTERVIEWER FILL OUT AT BEGINNING OF INTERVIEW

1.	Time interview started	☐☐Hrs ☐☐Min

INTERVIEWER FILL OUT AT THE END OF INTERVIEW

2.	Time interview ended	☐☐ Hrs ☐☐ Min
3.	Location of the interview	1. Block office<br>2. HSC<br>3. -87 Other (Specify) ________________
4.	Which language did the respondent speak? (circle the response)	1. Hindi<br>2. Odiya<br>-87 Other (specify) _________________
5.	Who was present at the interview? *(List all the people including the interviewer and the note taker)*	
6.	What is your assessment of the respondent's accuracy?	1. Good / okay<br>2. Not so good / bad (provide comments)
**Interviewer Comments**		

**Interview Status (Circle the appropriate choice)**	
**Completed**	**Respondent unavailable**
**Not found in the village**	**Respondent temporarily unavailable**
**Refused part way**	**Refused full interview**
**Recorded** (Yes) (No)	

**FOR SUPERVISOR ONLY**	**Date (dd/mm/yyyy)**					**Name of the supervisor**
Supervisor reviewed the interview						

Instructions: ***Where multiple choices exist (AWW/ANM/ASHA), CIRCLE the appropriate type of respondent you are directing the questions.***

[*italics*] = instructions to interviewer
(*italics*) = examples or concepts for the interviewer to keep in mind

A. GENERAL INFORMATION

1.	How long have you been working as ______________?
2.	What was your previous job/position?

B. WORKLOAD

3.	How much time do you spend every day working as (AWW/ANM/ASHA)?
4.	In the past 1 year, what tasks other than your regular work did you do because your supervisors told you to?<br><br>*[Probe:]*<br>• How often?

C. ICDS AND HEALTH SERVICES AND COORDINATION

5.	What services do <u>you</u> provide for mothers and children <2 years?<br><br>*[If not mentioned, probe for:]*<br>1-Counseling during home visits on breastfeeding<br>2-Counseling during home visits or community discussions on complementary feeding, including food safety, hygiene and sanitation<br>3-Maternal care and nutrition (for pregnant and lactating women)<br>4-IFA supplementation for 6-36 months and reproductive-age women<br>5-Immunization<br>6-Vitamin A supplementation<br>7-Deworming<br>8-Diarrhea treatment and prevention (ORS)<br>9-SAM treatment<br>10-Malaria treatment and prevention (bed nets)<br>11-Growth monitoring<br>12-Antenatal care<br>13-Supplementary nutrition for mothers and children
6.	How is ______(service)________ provided?<br><br>*[Probe:]*<br>• Ways (AWW/ANM/ASHA) is involved
7.	Which of these services require close coordination with (AWW/ANM/ASHA)?

8. Among these, where do you work well with (AWW/ANM/ASHA)? *[Ask for each other type of FLW.]*

   [Probe:]
   - What makes you say that it is well/good? *(definition of good coordination)*
   - Tell me about how you work together, from planning to service delivery.
   - What helps you in working together? *(facilitators to coordination)*
   - What makes it difficult to work together? *(barriers to coordination)*
   - Are there any guidelines on how to coordinate with (AWW/ANM/ASHA)?

9. In which service do you not work closely with (AWW/ANM/ASHA)? *[Ask for each other type of FLW.]*

   [Probe:]
   - What makes you say that it is not closely/poor? *(definition of poor coordination)*
   - Could you tell me about what happened in those situations?
   - Did you work together for this service before?
   - What has makes it difficult to work together? *(barriers, e.g. time, personal issues)*
   - Did you try (doing) anything to make the situation better?
   - Are there other instances where you faced poor coordination?

D. SPECIFIC MECHANISMS OF CONVERGENCE

10. *[If home visits for IYCF counseling is not yet identified]* How do you coordinate with (AWW/ANM/ASHA) for IYCF counseling?

    [Probe:]
    - How do you plan together for home visits?
    - Anything that helps/makes it difficult to coordinate? *(facilitators and barriers)*
    - Any guidelines on how to coordinate for home visits?

11. *[If VHND is not yet identified]* How do you coordinate with (AWW/ANM/ASHA) for VHND?

    [Probe:]
    - How do you plan and conduct VHNDs together?
    - Anything that helps/makes it difficult to coordinate? *(facilitators and barriers)*
    - Any guidelines on how to coordinate for VHNDs?

12. Tell me about the trainings that you received in the past one year.

    [Probe}
    - What were the topics of the trainings?

      [If not mentioned, probe for:]
      1-Breastfeeding counseling
      2-Counseling or community discussions on complementary feeding, including food safety, hygiene and sanitation
      3-Maternal care and nutrition (for pregnant and lactating women)
      4-IFA supplementation for 6-36 months and reproductive-age women
      5-Immunization
      6-Vitamin A supplementation
      7-Deworming
      8-Diarrhea treatment and prevention (ORS)

9-SAM treatment
10-Malaria treatment and prevention
11-Growth monitoring

- Duration
- Who provided
- Participants (AWW/ANM/ASHA)

[If no joint trainings:]

- Do you think there should be joint trainings?
- What would be helpful in making this possible?

13. How do you participate in <u>sector meetings</u> with (AWW/ANM/ASHA)?

[Probe:]

- Last time you attended a sector meeting with (AWW/ANM/ASHA)?
- Topics discussed
- Who provided
- Participants

[If not attended:]

- What are the reasons for not attending sector meetings with (AWW/ANM/ASHA)?
- What would be helpful in making this possible?

14. How do you participate in Village Health Sanitation Committee (VHSC) (meetings)?

[Probe:]

- Are you a member of VHSC?
- Last time you attended a VHSC meeting?
- Topics discussed
- Participants

[If not attended:]

- What are the reasons for not attending VHSC meetings?
- What would be helpful in making this possible?

15. How do you think coordination with (AWW/ANM/ASHA) influences service delivery? *(e.g. more efficient, increase coverage, or no changes)*

16. What are your thoughts about how the overall coordination with (AWW/ANM/ASHA) is working? *(e.g. helpful, not always practical or feasible, time consuming, not needed)*

[Probe:]

- Any suggestions on ways to improve the coordination?

E. ROUTINIZATION OF VILLAGE HEALTH AND NUTRITION DAYS (VHNDs)

17. Tell me how VHNDs started your village.

[Probe:]

- When did it start?
- What was implemented before VHND?
- Reason for starting VHND
- Any guidelines for VHND
- Is VHND written anywhere as part of your work responsibility? *(e.g. job description)*

18. Please tell me about how VHNDs are planned and conducted.

[Probe:]

- Who initiates the planning?
- How often does planning take place?
- What types of guidelines/manuals used? [*If available in written form, please ask to show it.]*
- Time usually spent in planning for and conducting VHND? How do you handle it?
- Where are VHNDs held?
- Who usually attends? *(e.g. pregnant, lactating women, children, adolescents, newly married couples)*
- Who else should be there but do not attend? Reasons that they do not attend?
- Any special order/way in how activities are conducted?
- Any rituals established with VHNDs? *(e.g. ceremonies, celebrations, periodic gatherings)*
- Reasons for conducting these rituals?

19. How did you learn about conducting VHNDs?

[*Probe:]*

- Training/orientation received? *(what was learned)*
- Last time that you received training/orientation?
- Who provided training/orientation?
- Other participants

- What services are usually provided during the VHNDs?

[If not mentioned, probe for:]

1-Breastfeeding counseling
2-Counseling or community discussions on complementary feeding, including food safety, hygiene and sanitation
3-Counseling on maternal care and nutrition (for pregnant and lactating women)
4-IFA supplementation for 6-36 months and reproductive-age women
5-Immunization
6-Vitamin A supplementation
7-Deworming
8-Diarrhea treatment and prevention (ORS)
9-SAM referrals
10-Malaria treatment and prevention (bed nets)
11-Growth monitoring
12-Registration of pregnant women
13-Antenatal care
14-Supplementary nutrition provided for underweight children

[Probe:]

- How often are these services provided during VHNDs? *(frequency of service)*
- How much time is spent on providing these activities during VHNDs? How do you handle it?

20. To what extent is money used to conduct VHNDs?

[Probe:]

- Different uses of money in conducting VHNDs? *(e.g. incentives for AWW or ASHA, preparation of complementary food, THR, etc.)*
- Where does the money come from? *(source of money)*
- Is the money usually enough to cover the needs? How do you handle it?
- For how long do you think that these funds will be available?

21. Tell me about the materials or equipment used for VHNDs.

[Probe:]

- Types of materials or equipment are required to conduct VHNDs? *(e.g. weighing scales, table, screen, medical supplies, THR, referral slips, IEC materials for counseling, MCPC cards, vaccinations, IFA tablets, deworming tablets, etc.)*
- Where do they come from? *(source of materials and equipment)*
- Are there usually enough? *(e.g. enough IFA tablets for all pregnant women attending VHND)*
- Condition of the materials and equipment? *(e.g. weighing scales calibrated)*
- How often are the materials or equipment available during VHNDs? *(e.g. vaccines, IFA always available)*
- For how long do you think that the materials or equipment will be available?
- Any difficulties in making the materials or equipment available?
- What helps in making the materials or equipment available?

22. How do people recognize VHND or talk about it?

[Probe:]

- Any visible logos, images or pictures that are commonly used to represent VHND?
- Any specific names or words for VHNDs? *[especially if VHND called by a different name]* Any words or phrases commonly used during VHNDs?

23. How is the VHND generally supervised?

[Probe:]

- Who from ICDS and health/NRHM supervises VHNDs?
- Who visited during the last VHND?
- What is usually done/checked when VHNDs are visited?

24. Tell me about any changes that you made to the VHNDs since it first started.

[Probe:]

- Types of changes made for the staff working on VHNDs? *(e.g. VHND task assignment to different staff involved)*
- Types of changes made to the facility, materials or equipment for VHNDs? *(e.g. painting of AWCs, new IEC materials, new equipment, etc.)*
- Any changes made to the types of activities conducted at VHNDs?
- Any changes made to how activities are organized or conducted?
- Reasons for making these changes? *[Probe for each of the changes.]*

[If no changes made:]

- What are some changes you would like to make to VHNDs?

- What keeps/stops you from making any changes?

25. In what ways do you think VHND helps or not helps you in delivering the services?

[Probe:]
- Ways service delivery improved because of VHND? *(e.g. quality, organization, coverage)*
- Ways service delivery got worse because of VHND?
- What has improved in your work because of VHNDs?
- What has become difficult in your work because of VHND?
- Any quality of service changes?

[If no changes:]
- Reasons for no changes?
- What needs to be done to make improvements?

26. How do you think VHND is influencing utilization of services?

[Probe:]
- Ways service utilization improved because of VHND? *(e.g. coverage, access, timeliness)*
- What helped to make such improvements?
- Ways service utilization got worse because of VHND?
- What difficulties led to such deterioration?

[If no changes:]
- Reasons for no changes?
- What needs to be done to make improvements?

F. WORK SUPERVISION

27. Who is your immediate supervisor?

28. Tell me about the meetings with your supervisor.

[Probe:]
- Purpose of the meetings? *(topics covered)*
- In the last 6 months, how many times did your supervisor visit you?
- Purpose of your supervisor's last visit?
- Who else usually comes with your supervisor when he/she comes to visit you?
- When do expect to meet your supervisor again?

G. JOB MOTIVATION AND SATISFACTION

29. What motivates you to work as an (AWW/ANM/ASHA)?

[Probe for:]
- Salary or monetary incentives

30. How do you feel about working as an (AWW/ANM/ASHA)?

[Probe:]
- What makes you feel this way?

- When do you feel this way? *(e.g. specific situations or events)*

31. To what extent do you receive support from your superiors *(i.e. block-level)*?

[Probe:]

- Which of your superiors provide support to you?
- Types of support received
- Other types of services that would help you

[If no support:]

- Types of support you would like to receive

32. To what extent do you receive support from the community or other groups?

[Probe:]

- Groups that provide support to you (*e.g. NGOs, mothers' groups)*
- Types of support received
- Other types of support that would help you

[If no support:]

- Types of support you would like to receive

33. Are there any ways to improve how you do your work?

[Probe:]

- Types of changes needed to improve your work
- Have you tried to make those changes?

[If no to above:]

- Reasons for not making such changes? What is keeping/stopping you from making such changes?

DEMOGRAPHIC INFORMATION

Lastly, I would like to ask you some information about your background.

34. Could you please tell me your age?	About ____________ years<br>88. Don't know / Do not want to tell
35. What is the highest level of schooling you have completed and passed?<br><br>[*Count only up to the level <u>passed</u>.*]	Did not complete Class 1<br>Class 1<br>Class 2<br>Class 3<br>Class 4<br>Class 5<br>Class 6<br>Class 7<br>Class 8<br>Class 9<br>Class 10<br>Class 11<br>Class 12<br>Graduate and above<br>Other diploma<br>Madrassa (if no formal classes exist)<br>Tuitions / teaching at home<br>Other training / courses / camps only<br>No schooling<br>-87 Other (specify)_______________
36. Are you presently engaged in any other work for which you receive compensation?	1. Yes<br>2. No

Thank you.
